# Reply to Dupuy et al. Comment on “Scaglioni et al. Present and Future of Autologous Breast Reconstruction: Advancing Techniques to Minimize Morbidity and Complications, Enhancing Quality of Life and Patient Satisfaction. *J. Clin. Med.* 2025, *14*, 2599”

**DOI:** 10.3390/jcm15020601

**Published:** 2026-01-12

**Authors:** Mario F. Scaglioni, Federica Martini, Matteo Meroni

**Affiliations:** 1 Plastic Surgery Pyramide, Haus zur Pyramide, 8008 Zurich, Switzerland; federicaa.martini@gmail.com (F.M.); matteomeronimd@gmail.com (M.M.); 2 Department of Health Sciences and Medicine, University of Lucerne, 6005 Lucerne, Switzerland; 3 Department of Hand- and Plastic Surgery, Luzerner Kantonsspital, 6000 Lucerne, Switzerland; 4 Department of Plastic, Reconstructive and Aesthetic Surgery, University of Milan, 20122 Milan, Italy

We would like to sincerely thank Dr. Dupuy and co-authors for their thoughtful and constructive comments [1] on our article [2], recently published in the *Journal of Clinical Medicine*.

We are very pleased that you share our interest in the ongoing development of autologous breast reconstruction and acknowledge the importance of the technical innovations we discussed. At the same time, we completely agree with the core message of your letter: real innovation in autologous breast reconstruction is about more than just technology.

As emphasized, the organization of care plays a crucial role, since large parts of the surgery and outcome are determined before the procedure through thorough preoperative planning together with the breast surgeon. In fact, structured planning, tailored to the patient’s anatomical features, oncological requirements, and personal expectations, is a vital part of the reconstructive process.

We appreciate your focus on reducing the number and complexity of surgical stages, such as immediate contralateral symmetrization, careful primary placement of the abdominal scar, and flap shaping to closely match the final outcome during the initial procedure. This approach aligns with our discussion on the importance of minimizing “reconstructive burnout” [3] by avoiding multiple small secondary procedures whenever it is safe to do so. Additionally, your experience with fully de-epithelialized, buried flaps in both immediate and delayed settings, showing low complication rates, is a valuable and encouraging complement to the evidence we cited.

Moreover, as you correctly emphasize, Enhanced Recovery After Surgery (ERAS) protocols [4], including comprehensive preoperative counseling, optimized imaging, day-of-surgery admission, morphine-sparing anesthesia, regional blocks, early mobilization, and discharge within a few days, play a crucial role. Our review also highlights ERAS as a vital element of modern autologous reconstruction, underlining its benefits in shortening hospital stays, lowering complication rates, and enhancing physical and psychological recovery. Your detailed description of a high-efficiency workflow, such as standardized room organization, simultaneous flap harvest and mastectomy, and double-team operating, clearly illustrates how these principles can be implemented in daily practice, highlighting the connection between organizational refinement and technical innovation. Structured organization during surgery and postoperative care improves efficiency, ultimately reducing operative time and complications [5].

Regarding ICG angiography, you mention that, in your experience, routine use might extend operative time, interfere with workflow, and provide only limited additional benefit beyond careful clinical assessment of dermal bleeding. However, in our practice we find ICG a valuable tool in flap reconstruction, particularly when used systematically at the following key stages: after flap elevation, after microsurgical anastomosis, immediately after flap inset, and after wound closure.

When used this way, it helps us to identify when and where a complication occurs. For instance, if ICG at the end of flap elevation, before clipping major vessels, shows impaired perfusion, it suggests that the problem started during dissection. On the other hand, if the flap remains brightly fluorescent after microsurgical anastomosis, it indicates venous congestion and requires targeted correction. Thus, ICG does not replace clinical judgment but provides additional objective information, which could also be useful for the medical record, to objectively demonstrate the procedure’s functionality during surgery, and to reduce uncertainty among surgeons in borderline situations. We also find it especially useful in training, where it helps less-experienced surgeons assess flap vitality and avoid errors. In our experience, its use has been associated with lower flap loss rates, fewer re-explorations, and improved overall efficiency [6]. However, we see these approaches as complementary rather than conflicting and believe the choice should be individualized based on the surgeon’s experience and preferences.

We greatly appreciate your final emphasis on training the next generation of surgeons through structured mentorship and progressive autonomy. The concept of “surgical companionship” is particularly relevant in a field such as autologous breast reconstruction, where procedures are complex, technically demanding, and associated with a long learning curve. A stepwise progression, from initial observation to performing selected steps under close supervision and finally to carrying out the entire procedure with senior support, provides a safe and effective framework for skill acquisition. We believe senior surgeons have a significant responsibility towards their younger colleagues. In the current landscape, it is no longer acceptable to assume that the next generation will “learn by osmosis” just by observing and assisting senior surgeons. Similarly to how one cannot learn to drive a car solely by watching others, even for many years, it is unrealistic to expect trainees to perform complex microsurgical reconstructions safely and effectively without structured, hands-on training and gradual trust. Therefore, it is our duty to actively teach, clarify our decision-making processes, share technical details, and offer supervised chances for independent practice. From our experience, this intentional transfer of skills helps make these procedures more reproducible and accessible for younger surgeons, ultimately benefiting future patients and promoting the discipline’s long-term growth.

However, the primary aim of our narrative review was to highlight recent technical and technological developments that are reshaping the way surgeons plan and perform autologous breast reconstruction, moving progressively toward less invasive and more refined procedures. By improving flap design, intraoperative assessment, and perioperative management, these innovations provide surgeons with concrete tools that when incorporated into standardized protocols and applied within a structured clinical framework can enhance operative efficiency, reduce morbidity, and ultimately improve the quality and accessibility of reconstructive care. At the same time, we fully recognize that even a surgeon equipped with the most advanced robotic systems and technologies does not automatically achieve better or safer results; their real value depends on appropriate indications, good judgment, and thoughtful integration into well-organized care pathways.

In summary, we appreciate Dr. Dupuy and colleagues for their thoughtful and constructive comment which significantly broadens the discussion by highlighting the importance of teamwork, workflow, and training, elements which are often less visible than technical or technological advances but equally essential. By illustrating how these organizational and educational factors enhance the safety, predictability, and accessibility of autologous reconstruction for more patients, your input complements our focus on technological innovation and contributes to a more comprehensive understanding of true progress in this field. We hope this exchange encourages further dialogue and collaboration among teams working, all aiming to provide more women with safe, effective, and accessible autologous breast reconstruction.
